# D-galactose Intake Alleviates Atopic Dermatitis in Mice by Modulating Intestinal Microbiota

**DOI:** 10.3389/fnut.2022.895837

**Published:** 2022-06-21

**Authors:** Dong-Yeon Kim, Do-Hyeon Jung, Eun-Jung Song, Ah-Ra Jang, Ji-Yeon Park, Jae-Hun Ahn, Tae-Sung Lee, Yeong-Jun Kim, Yun-Ji Lee, In-Su Seo, Hye-Eun Kim, Eun-Ju Ryu, Jaehyun Sim, Jong-Hwan Park

**Affiliations:** ^1^Laboratory Animal Medicine, College of Veterinary Medicine and Animal Medical Institute, Chonnam National University, Gwangju, South Korea; ^2^Quorum Bio Co., Ltd., School of Dentistry, Seoul National University, Seoul, South Korea

**Keywords:** D-galactose, atopic dermatitis, inflammation, microbiota, nutrition

## Abstract

Atopic dermatitis (AD) is one of the most prevalent, chronic and persistent inflammatory skin diseases closely associated with intestinal microbiota. To evaluate the effect of D-galactose intake on AD, we orally administered D-galactose to BALB/c mice whose ears and skin were treated with 2,4-dinitrochlorobenzene (DNCB). D-galactose alleviated DNCB-induced AD-like phenotypes such as redness, scaling/dryness and excoriation. Ear thickness was also decreased by D-galactose administration. Histopathological analysis revealed decreased epidermal thickening, infiltration of immune cells, especially mast cells, in the dermis. Total levels of serum IgE representing the immunological response of AD were decreased by D-galactose administration. Microbiota analysis showed that D-galactose administration restored gut microbiota profiles, which were altered in AD mice, characterized by increased abundance of Bacteroidetes and decreased abundance of Firmicutes. The increased abundance of Bacteroides and the decreased abundance of Prevotella and Ruminococcus were reversed by D-galactose treatment, following improvement of AD. Our results suggest the possible use of D-galactose as a prebiotic to alleviate AD by altering gut microbiota.

## Introduction

Atopic dermatitis (AD) is a chronic inflammatory skin disease, which is characterized by relapsing eczematous, oozing and weeping pruritic symptoms over dry skin with epidermal hyperplasia (1). AD affects individuals worldwide, especially children, with increased prevalence in the last few decades (2, 3). The pathogenesis of AD involves a complex interplay between genetic risk factors, epidermal barrier dysfunction, skin microbiota abnormalities, and immunological dysregulation. The management of AD is still a challenge in clinical practice, which focuses on symptomatic treatment. Most clinics have used corticosteroids as the first-line treatment, which may cause severe side-effects when used long term (4). Thus, there is a large demand for safer and effective treatments for AD compared with classical therapy.

Gut microbiota, that is the “virtual organ” play a key role in a variety of nutritional, metabolic, physiological, and immunological processes while maintaining homeostasis. Dysbiosis, an imbalance in microbial homeostasis, is associated with the incidence of several diseases (5, 6). In addition, gut dysbiosis leads to a rise in the prevalence of AD in terms of the Gut-skin axis by disturbed systemic immune balance resulting in alteration of the integumentary system or translocation of metabolites from the gut microbiota and its components in dysbiosis (7–9). For instance, the proportions of Bacteroidetes, *Bifidobacteria* and *Bacteroides* are decreased in the gut microbiota of patients with AD than in healthy controls, while the concentrations of clostridia*, E. coli*, and *S. aureus* are increased in AD due to eosinophilic inflammation (8, 10–13). *R. gnavus* is associated with alleviation of AD mediated *via* enhanced regulatory T-cell counts and SCFAs production (14). Lee et al. reported that the reduction in the colonization of *R. gnavus, A. muciniphila*, and *Lachnospiraceae* spp. occurs in immune-related gene expression, which is associated with stunted immune development in infants with AD compared with the control group (15). Moreover, some studies have demonstrated that gut microbiota can have a positive impact on the response to the disruption of the skin barrier. Administration of *L. helveticus* reduced the disruption of the skin barrier that was induced by SDS (sodium dodecyl sulfate) and promoted the epithelial barrier function (16). The gut microbiota can influence the skin microbiota as well. For instance, Propionibacterium is a genus that produces SCFAs, especially propionic acid. Propionic acid has a strong antimicrobial effect on USA300, the community-acquired MRSA which is known as the main pathogen of AD. Considering all these reports, it suggested that modulation of gut microbiota can be used to manage AD. Given the integral role of microbiota in immune development and homeostasis, the modulation of microbiota may be useful in both the treatment and prevention of allergic disorders including AD *via* epithelial, immunological, and microbial effects (9, 17).

D-galactose is a monosaccharide sugar derived primarily from dairy products. D-galactose is normally transformed into hepatic glycogen to facilitate whole-body glucose utilization and brain function (18, 19). The intake of D-galactose altered the intestinal microbiota profile (20, 21). Diets containing D-galactose improved hepatic insulin sensitivity by decreasing the ratio of Firmicutes:Bacteroidetes by 70% compared with glucose and fructose in SD rats (20). Galacto-oligosaccharides (GOS) consist of multiple galactose chains and are widely available commercially as prebiotics. GOS are also associated with the prevalence of AD. In a prospective, placebo-controlled trial, prebiotic mixtures comprising 90% GOS and 10% FOS showed benefits in AD of infants (22). Kuitunen et al. (23) also reported that a mixture of four probiotics and GOS significantly prevented the incidence of atopic eczema by age 2 years. However, the relationship between oral intake of D-galactose and the pathogenesis of AD has yet to be elucidated based on altered gut microbiota.

Here, we investigated whether the oral intake of D-galactose ameliorated clinical symptoms of 2,4-dinitrochlorobenzene (DNCB)-induced AD-like skin disease in a mouse model. We validated our hypothesis that D-galactose has a protective effect on AD by inducing changes in gut microbiota including altered microbial richness and diversity, and in specific bacterial species associated with AD.

## Materials and Methods

### Animals

Seven-week-old male mice on a BALB/c background were purchased from Damul Science (Daejeon, Korea), followed by acclimation for 1 week. The animals were housed in a standard laboratory animal room at 21 ± 2°C and 50 ± 5% humidity and maintained under a strict 12/12 h light/dark cycle. Sterilized feed and water were provided *ad libitum*. Animal experiment was approved by the Institutional Animal Care and Use Committee of Chonnam National University (Gwangju, Korea; approval no. CNU IACUC-YB-2020-68).

### *In vivo* Experiment

**Figure 1:**
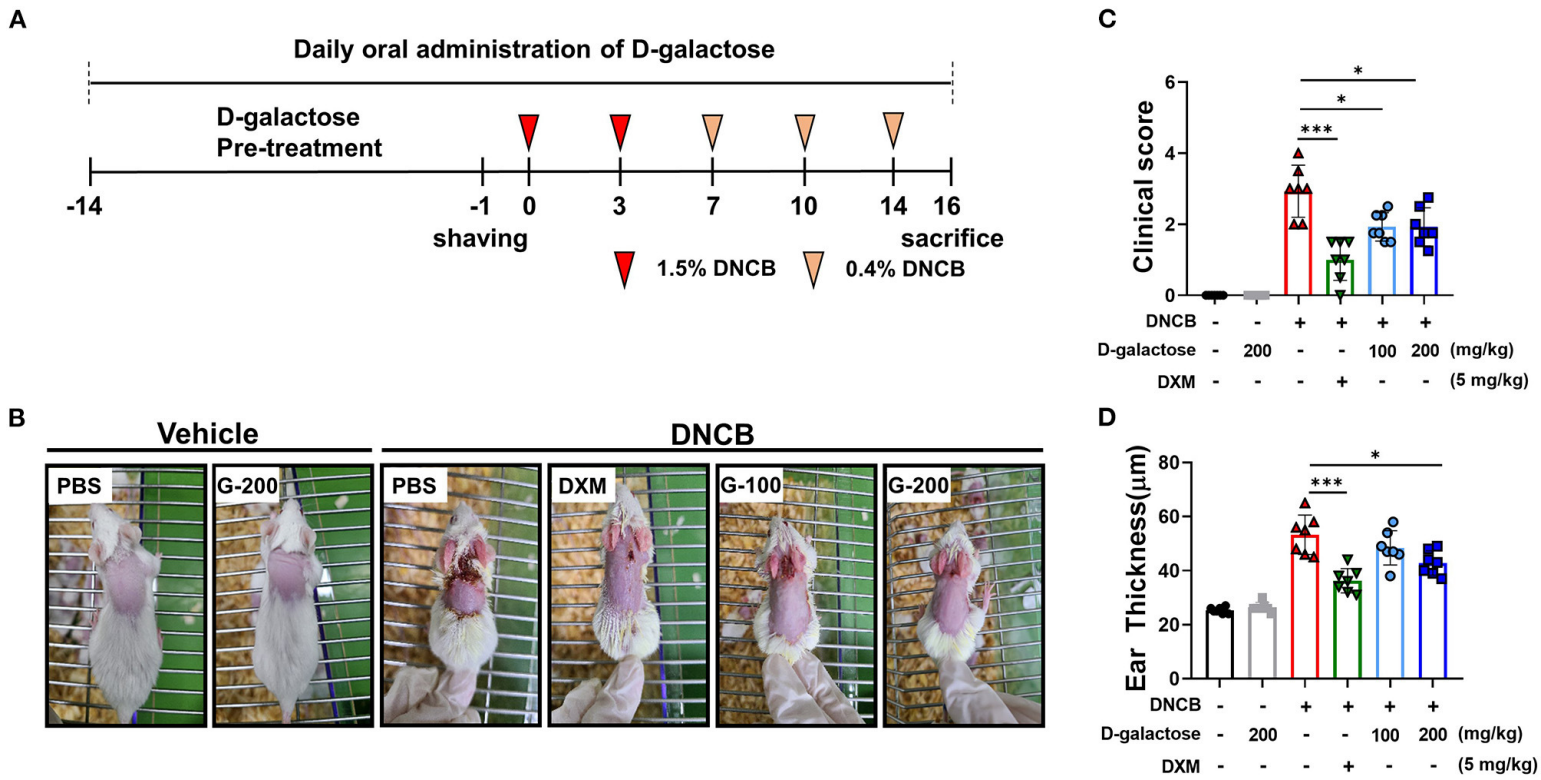
Protective effects of D-galactose on DNCB-induced atopic dermatitis in mice. **(A)** Schematic diagram showing the experimental design of DNCB-induced AD mouse model. Mice were divided to six groups (*n* = 7/group). **(B)** AD-like skin lesions were evaluated by visual observation. **(C)** The dermatitis index was scored as described in the materials and methods. **(D)** Ear thickness was measured before sacrificing. Values are means ± SD. The significance of differences between the groups was assessed using Mann-Whitney *U*-test, with the level of significance set at *P < 0.05, **P < 0.01, ***P < 0.001.

For the experiment, a total of 42 mice were used in the experiment. The 42 mice were divided into six groups with seven mice in each group: (1) a vehicle group, (2) vehicle + D-galactose group, (3) DNCB-treated group (negative control), (4) DNCB-treated + dexamethasone (DXM, 5 mg/kg; positive control), (5) DNCB-treated + D-galactose (100 mg/kg) groups, and (6) DNCB-treated + D-galactose (200 mg/kg) groups. DNCB was dissolved in an acetone: olive oil mixture (3:1). D-galactose was obtained from Quorum Bio Co., Ltd., Seoul, South Korea. DXM was injected i.p. Before inducing dermatitis, D-galactose was orally administrated daily for 2 weeks. The dorsal hair of mice was shaved using clippers with hair removal cream, followed by application of 200 μl of 1.5% DNCB to the shaved dorsal skin and 50 μl to the ears on days 0 and 3 (sensitization), and 0.4% DNCB to the dorsal skin (200 μl) and ears (50 μl) on days 7, 10, and 14 (challenge). At the end of the experiment, the fecal samples were collected to evaluate the microbial composition. Animals were sacrificed and subjected to histopathological analysis. The serum samples were collected to measure the total serum IgE level. The experimental schedule was depicted in Figure 1A.

### Skin Dermatitis Scoring and Measurement of Ear Thickness

The clinical severity of DNCB-induced dermatitis was evaluated twice a week. The symptoms were evaluated based on (1) erythema/hemorrhage, (2) dryness/scaling, and (3) excoriation scored as 0 (none), 1 (mild), 2 (moderate), or 3 (severe). The sum of the individual scores was determined by the dermatitis clinical score. Ear thickness was measured with a micrometer (Mitutoyo, Kawasaki, Japan) on the day of sacrifice. The micrometer was used at the same spot of the ear except for the cartilaginous ridges. To minimize technical variation, a single researcher conducted the measurement during each experiment.

### Histological Analysis

After mice were sacrificed, the sliced ear and dorsal skin tissues were fixed in 10% neutral buffered formalin for 48 h. Tissues were embedded in paraffin, and the paraffin blocks were sectioned at 2 μm, followed by hematoxylin and eosin staining using standard techniques. We used toluidine blue to trace mast cell infiltration in dermis. Histopathological examination was performed under light microscopy. An arbitrary range was assigned to each microscopic field at 100× magnification. Histologic scoring was evaluated by two experts in laboratory animal pathology. The scoring system assesses the severity of skin lesions based on three features: epithelial hyperplasia, infiltration of inflammatory cells including mast cells and eosinophils in dermis, and degree of keratosis (dyskeratosis/parakeratosis). Each feature was scored on a scale of 0 to 3 (0: none, 1: mild, 2: moderate, 3: severe) and the aggregate of the scores was determined as a total score.

### Enzyme-Linked Immunosorbent Assay

Blood samples were collected prior to the sacrifice of the anesthetized mice. To identify the difference in total serum IgE production depending on the presence or absence of D-galactose administration in DNCB-induced AD, serum IgE (assay range 4–250 ng/ml) was subjected to ELISA according to the manufacturer's protocols.

### Extraction of Bacterial DNA From Mouse Stool

The bacterial DNA was extracted using a PowerMax Soil DNA Isolation Kit (MO BIO, Carlsbad, CA, United States) according to the described protocol. Briefly, the stool samples were lysed by homogenization. Lysis buffer was added to the sample. The crude lysate was then subjected to inhibitor removal for cleanup. The purified lysate was mixed with an equal volume of DNA binding solution and passed through a spin filter membrane. Then DNA was eluted using elution buffer. Each sample was prepared according to the Illumina 16S Metagenomic Sequencing Library protocols to amplify the specific (V3 and V4) region. The DNA quality was measured using PicoGreen and Nanodrop methods. A 10 ng gDNA sample was amplified with the following primer sequences: 341F: 5′-CCTACGGGNGGCWGCAG-3′, 806R: 5′-GACTACHVGGGTATCTAATCC-3′.

The final product was quantified using qPCR according to the qPCR Quantification Protocol Guide and qualified using the LabChip GX HT DNA High Sensitivity Kit (PerkinElmer, Waltham, MA, United States). The paired-end sequencing was performed using the MiSeq™ platform (Illumina, San Diego, CA, United States).

### OTU Analysis of MiSeq

Macrogen Ltd. performed the sequencing. Briefly, after completing the sequencing, MiSeq raw data was classified for each sample using an index sequence, and a FASTQ file for each sample was generated. The adapter sequence was removed and error-correction was performed on the region where the two read overlaps using fastp program. The obtained sequence was obtained by using CD-HIT-OTU, an OTU analysis program based on CD-HIT-EST, to remove low-quality sequences, ambiguous sequences, and chimera sequences. The sequence was clustered with similar sequences *via* a series of processes to form species-level OTU with sequence similarity of 97% or more. For the representative sequence of each OTU, BLAST+(v2.9.0) was performed with the Reference DB (NCBI 16S Microbial). Taxonomic assignment was performed with the organism information of the subject with the highest similarity. We analyzed in a set of samples on multiple taxonomic levels for total 11 phyla, 21 classes, 35 orders, 62 families, 148 genera, and 216 species. In addition, comparative analysis of various microbial communities was performed using QIIME (v1.9) based on the above OTU abundance and taxonomy information. To confirm the species diversity of the microbial community, Shannon Index and Inversed Simpson Index were obtained. The alpha diversity was confirmed using the Rarefaction curve and Chao1 value. In addition, beta diversity of samples was calculated based on the Weighted/Unweighted UniFrac distance.

### Statistical Analysis

All graph values are presented as mean ± SD. The statistical significance of differences between groups was determined using Mann-Whitney *U*-test in all tests; *P*-Values < 0.05 were considered statistically significant.

## Results

### Protective Effects of D-galactose on DNCB-Induced Atopic Dermatitis in Mice

AD-like skin lesions were induced by DNCB treatment in BALB/c mice as depicted in Figure 1A. On Day 16, the dorsal skins and ears of mice treated with DNCB showed remarkable AD-like lesions including erythema, excoriation, and scaling/dryness compared with the vehicle group, indicating that DNCB induced AD-like phenotypes in mice (Figure 1B). We found that treatment with dexamethasone (DXM) as a positive control reduced the severity of the dermatitis compared with the vehicle group (Figure 1B). Oral intake of D-galactose alleviated AD-like lesions in the 100 mg/kg and 200 mg/kg groups (Figure 1B). The AD clinical index decreased significantly in groups treated with D-galactose (100 and 200 mg/kg) as well as DXM, when compared with the PBS treated DNCB group (Figure 1C). Ear thickness was dramatically increased in DNCB-treated mice compared with vehicle group, which was also restored in groups treated with D-galactose (200 mg/kg) and DXM (Figure 1D).

### Effects of D-galactose on Spleen and Axillary Lymph Node on DNCB-Induced Atopic Dermatitis in Mice

**Figure 2:**
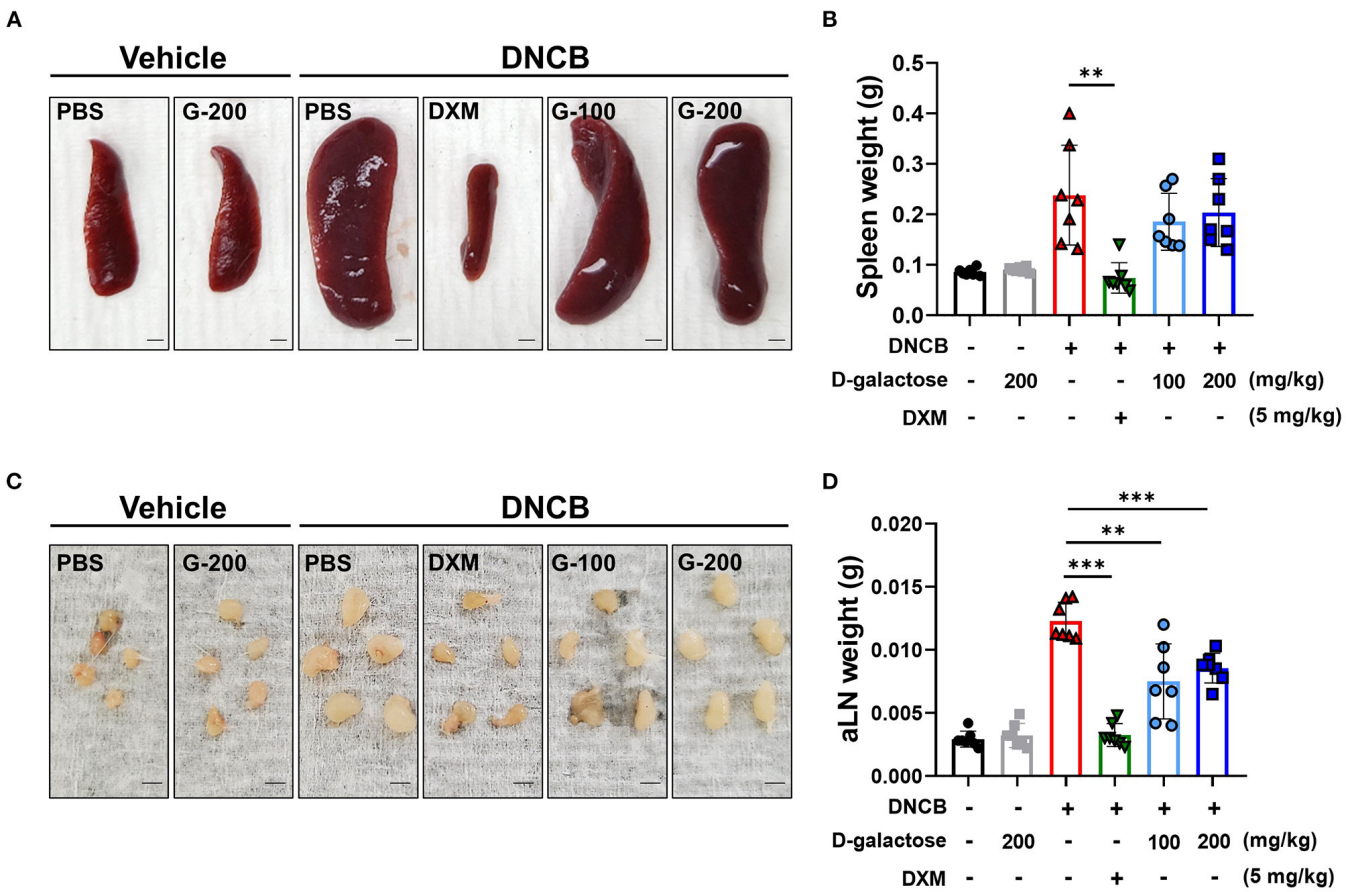
Effects of D-galactose on spleen and axillary lymph nodes of mice with DNCB-induced atopic dermatitis. Size comparison of **(A)** spleen and **(C)** axillary lymph node with or without D-galactose treatment (Scale bars, 1 mm). Comparison of **(B)** spleen weight and **(D)** axillary lymph node weight. Values are means ± SD. Significant differences between the data were assessed using Mann-Whitney *U*-test, with the level of significance set at *P < 0.05, **P < 0.01, ***P < 0.001.

To investigate whether D-galactose decreases systemic immune response in mice, we measured the size and weights of the spleen and axillary lymph nodes (24). Repetitive application of DNCB increased the size and weight of spleen and axillary lymph nodes in DNCB-treated mice, which was completely restored by DXM treatment (Figures 2A–D). The mean level of spleen weight was slightly lower in D-galactose-treated groups compared with PBS-treated DNCB group, although there was no significant difference (Figures 2A,B). In addition, D-galactose treatment resulted in a decrease in size and weight of the axillary lymph nodes significantly, although no dose-dependent effect was observed (Figures 2A–D).

### D-galactose Intake Decreases Serum IgE Level in DNCB-Induced AD Mice

**Figure 3:**
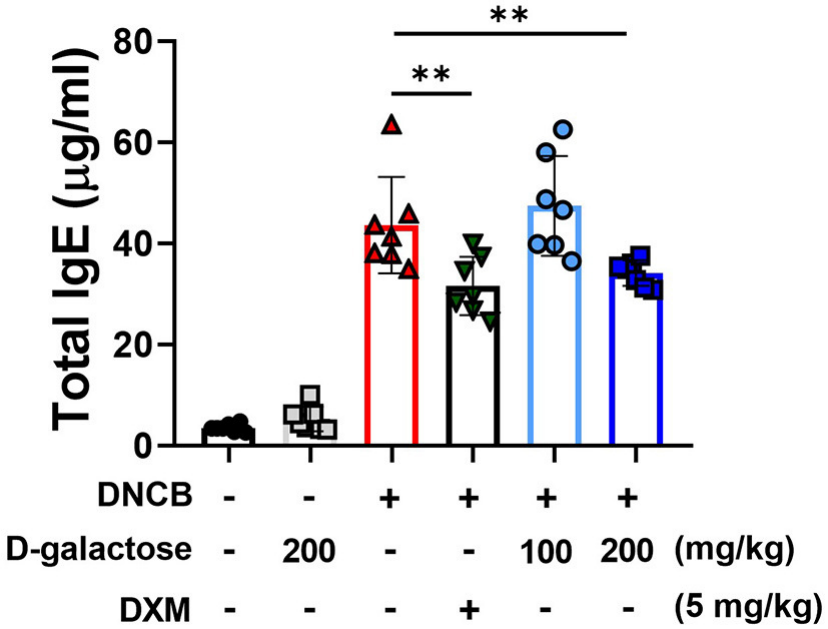
Effects of D-galactose on DNCB-induced serum IgE levels in mice. Blood was collected on the day of sacrifice in the experiment (day 14). The level of serum IgE was measured with ELISA. Values are means ± SD. Significant differences between the groups were assessed using Mann-Whitney *U*-test, with the level of significance set at *P < 0.05, **P < 0.01, ***P < 0.001.

Atopic dermatitis is a typical skin inflammatory disorder associated with dramatically increased levels of IgE and inflammatory cytokines (25). DNCB application increased the serum levels of total IgE, which was reduced by DXM treatment (Figure 3). Although D-galactose intake of 100 mg/kg did not alter the serum IgE level, the level was reduced in the group treated with 200 mg/kg of D-galactose (Figure 3). These findings suggest that D-galactose intake may alleviate AD-like lesions by regulating IgE production.

### D-galactose Intake Reduced DNCB-Induced Skin Lesions

**Figure 4:**
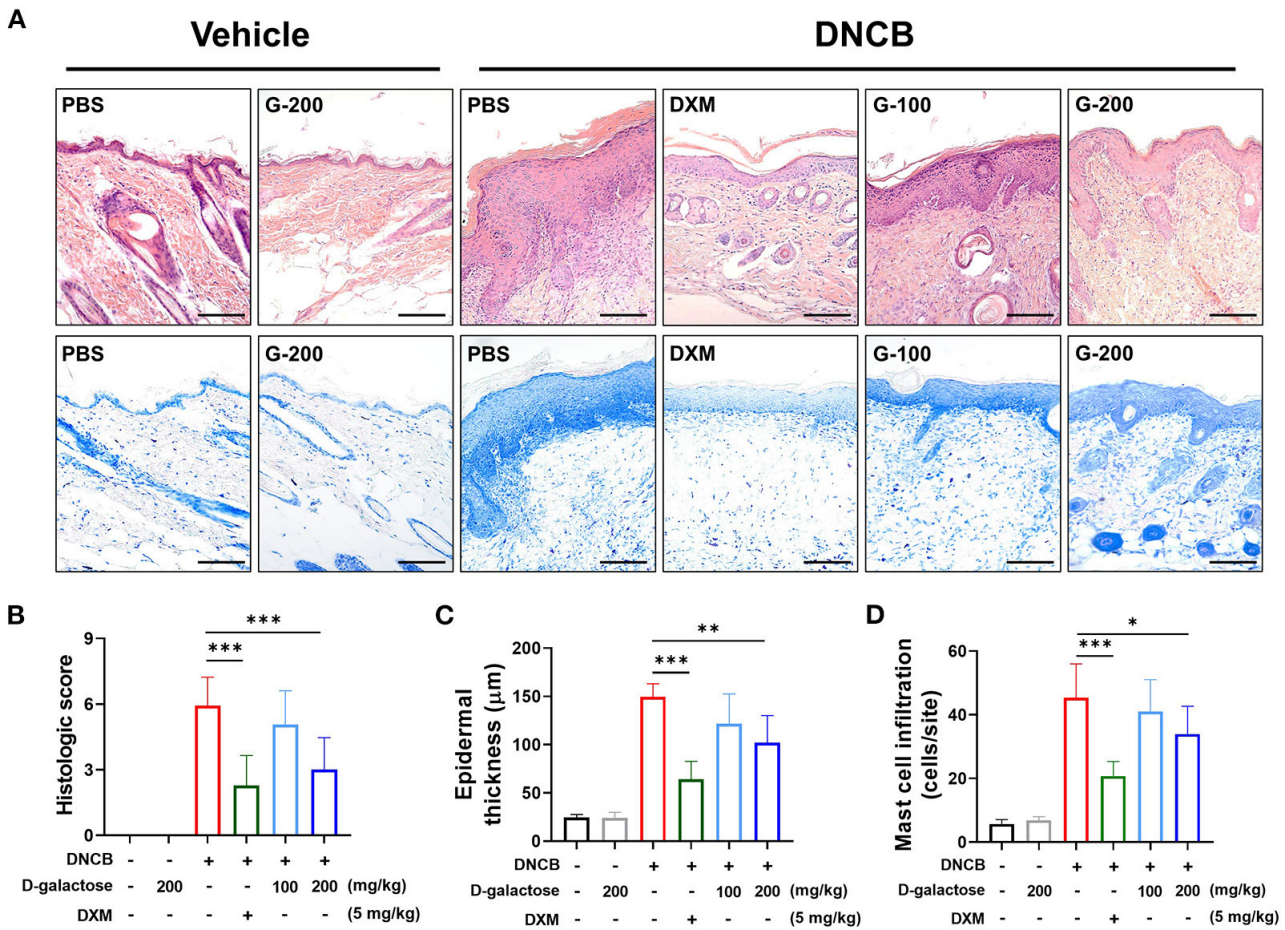
Effect of D-galactose on cutaneous histopathological observations of DNCB-induced atopic dermatitis in mice. Representative histological findings of cutaneous tissue sections stained with **(A)** hematoxylin and eosin or toluidine blue staining (Scale bars, 100 μm). Histological evaluation of **(B)** cutaneous tissue sections and **(C)** epidermal thickness. **(D)** Mast cell infiltration was quantified and compared among the groups. Values are means ± SD. The significance of differences between the group was assessed using Mann-Whitney *U*-test, with the level of significance set at *P < 0.05, **P < 0.01, ***P < 0.001.

We further investigated the effect of D-galactose intake on DNCB-induced skin lesions such as epithelial hyperplasia, infiltration of inflammatory cells composed of eosinophils and mast cells in dermis, degree of keratosis (dyskeratosis/parakeratosis), and epidermal or ear thickness. Histologic score was evaluated using the scoring system described Material & Method. We used H&E staining to identify the effects of D-galactose on skin lesions and infiltration of eosinophils and mast cells *via* Toluidine blue staining. The elevated histological score in dorsal skin of DNCB-treated mice was decreased by D-galactose intake in a dose-dependent manner (Figures 4A,B). Epidermal thickness was also increased by DNCB application. D-galactose intake reduced the thickness in a dose-dependent manner (Figures 4A,C). In addition, the increased infiltration of mast cells and eosinophils in dermis occurred in the dorsal skin of DNCB-treated mice, which was decreased by D-galactose intake in a dose-dependent manner (Figures 4A,D). In all cases, DXM treatment alleviated AD pathologic lesions as a positive control (Figures 4A–D). Likewise, ear skin was examined and histological analysis was performed. DNCB application increased the histologic score, which was reduced by DXM treatment and D-galactose intake of 200 mg/kg, whereas D-galactose intake of 100 mg/kg reduced the ear histologic score but not significantly (Supplementary Figures S1A,B). Ear epithermal thickness and mast cell infiltration was also significantly decreased with D-galactose intake (200 mg/kg) (Supplementary Figures S1C,D).

### Microbiota Modulate D-galactose Administration in DNCB-Induced AD

**Figure 5:**
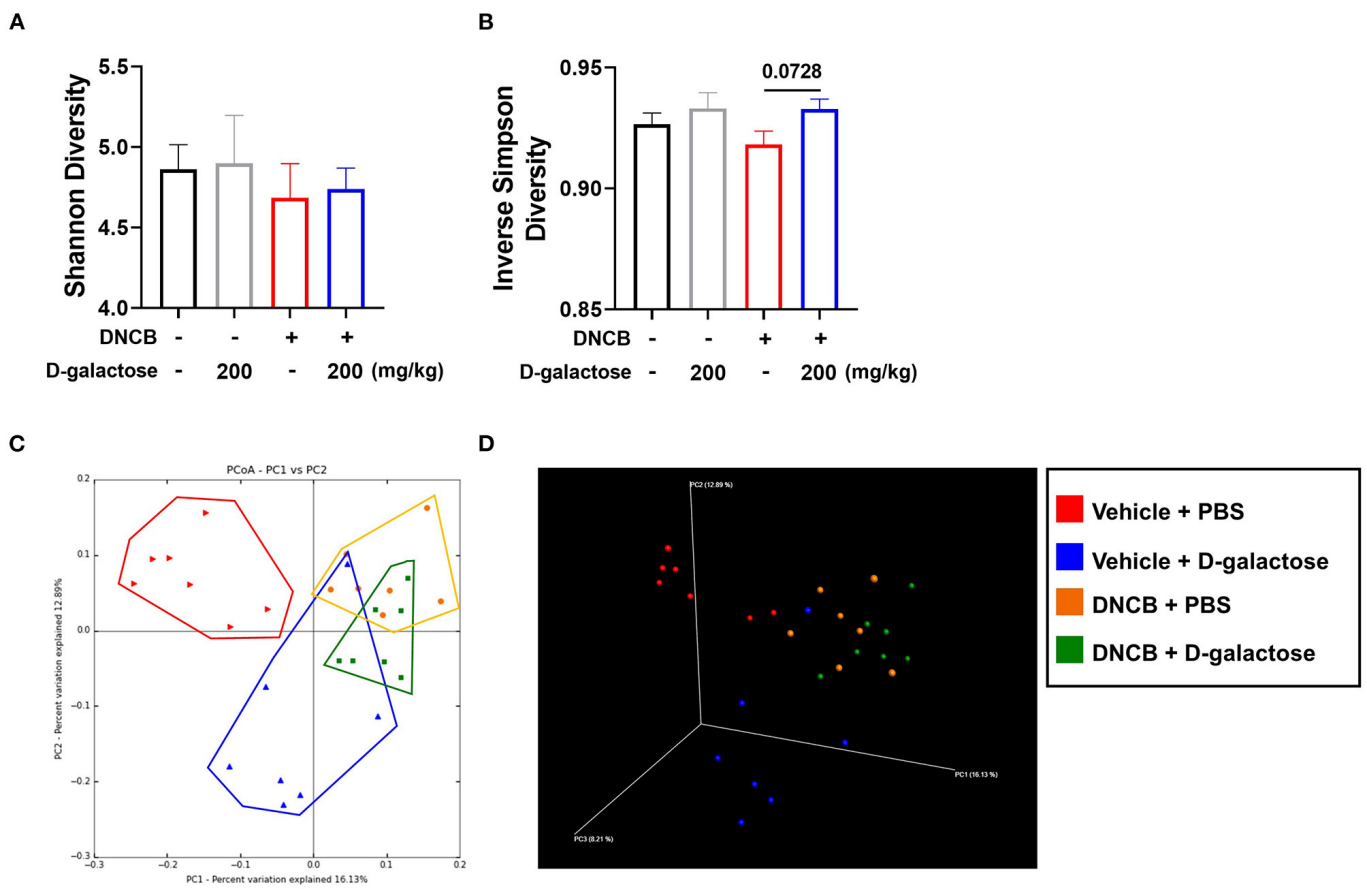
Effects of D-galactose on alpha and beta diversity. Alpha and beta diversity of intestinal microbiota in vehicle-treated and DNCB-treated mice with or without D-galactose treatment was evaluated. **(A,B)** Richness of intestinal microbiota in indicated by Shannon diversity and Inverse Simpson Diversity **(C,D)** Beta diversity 2D and 3D patterns were determined by principal coordinate analysis. PBS + Vehicle (Red), D-galactose + Vehicle (Blue), PBS + DNCB (Yellow), D-galactose + DNCB (Green). The significance of differences between the groups was assessed using Mann-Whitney *U*-test, with the level of significance set at *P < 0.05, **P < 0.01, ***P < 0.001.

To investigate the microbial environment in the mouse intestine, mouse stool was collected from each group before sacrifice. Several indices were used to evaluate the richness and diversity of intestinal microbiota. The Shannon index, which indicates the evenness of fecal microbiota, was lower in the DNCB-treated group than in the vehicle group. However, D-galactose treatment resulted in slightly higher Shannon index than in PBS-treated DNCB group (Figure 5A). The Inverse Simpson index was also higher after D-galactose treatment than in PBS-treated DNCB group (Figure 5B). However, other indices such as OTUs, Chao1, and Good's coverage diversity index did not show similar changes (Supplementary Figure S2). A beta diversity analysis using unweighted principal coordinate analysis (PCoA) revealed differences in intestinal microbiota composition among the four groups (Figures 5C,D). Results of weighted principal coordinate analysis are shown in Supplementary Figure S3.

**Figure 6:**
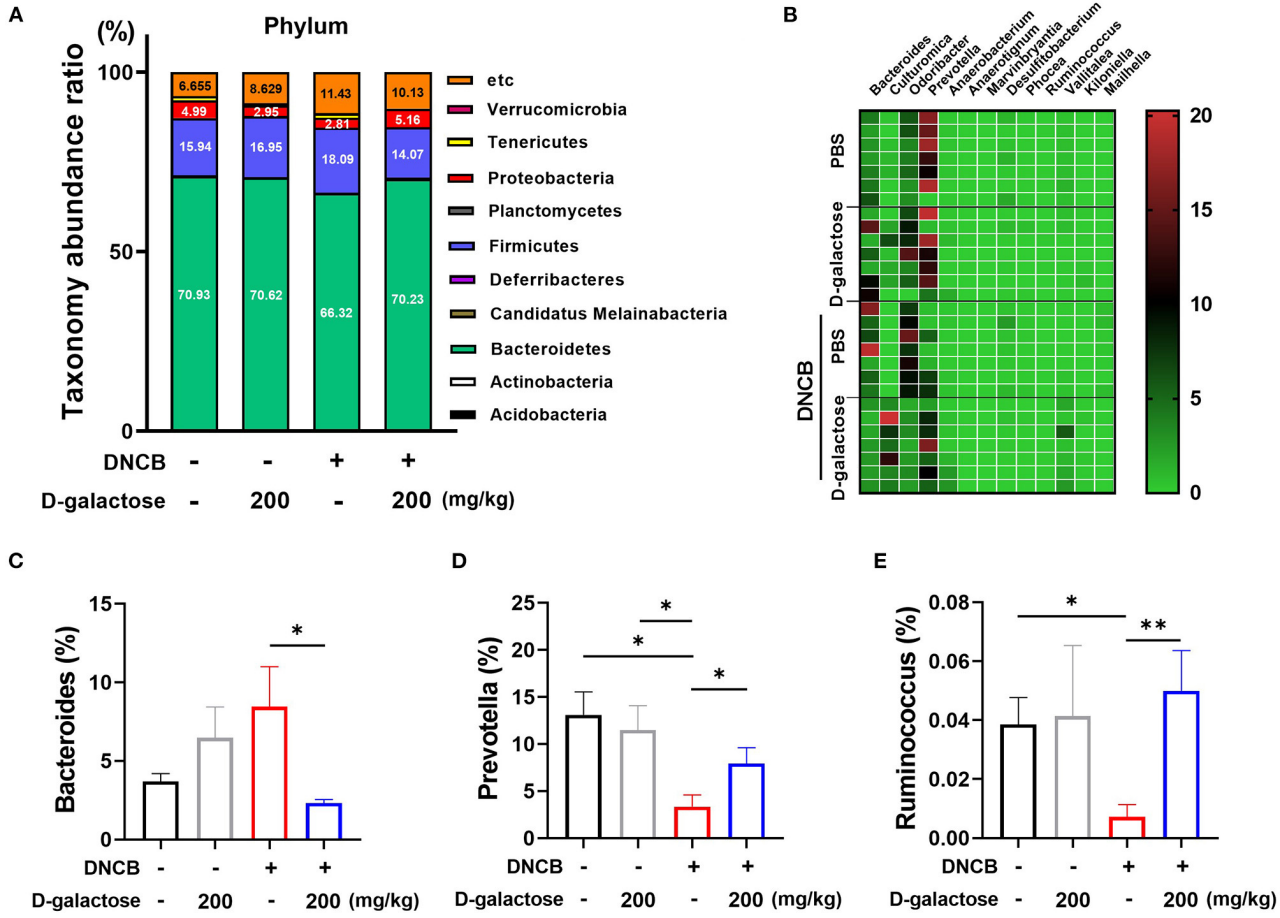
Effects of D-galactose on intestinal microbiota composition in mice with DNCB-induced atopic dermatitis. **(A)** Relative abundance of bacterial phyla in the intestinal microbiota of mice. The color of each cell indicates the relative abundance of bacterial phyla. **(B)** Relative abundance of intestinal microbiota at the genus level in mice, which showed a significant difference between DNCB-induced AD groups. **(C–E)** Relative abundance of *Bacteroides, Prevotella* and *Ruminococcus* among the intestinal microbiota of mice. Values are means ± SD. Significant differences between the groups were assessed using Mann-Whitney *U*-test, with the level of significance set at *p < 0.05, **p < 0.01, ***p < 0.001.

We analyzed the alterations in microbiota composition of each group at the phylum level. As shown in Figure 6A, there was no significant change between the D-galactose treated and untreated vehicle groups. However, the DNCB treatment resulted in changes in phylum level. Our data showed that the relative abundance of Bacteroidetes was decreased, whereas Firmicutes level was increased. However, oral administration of D-galactose restored the levels of Bacteroidetes and Firmicutes to levels in the vehicle group. The relative abundance of 13 genera showed significant differences based on D-galactose administration in AD mice treated with DNCB (Figure 6B). Within phylum Bacteroidetes, the levels of *Bacteroides* decreased significantly in the group treated with D-galactose (Figure 6C). However, the relative abundance of *Prevotella* decreased significantly in those groups (Figure 6D). Within phylum Firmicutes, *Ruminococcus* displayed a significant increase in mice with AD treated with DNCB and exposed to D-galactose (Figure 6E). In class, order, family, and other genus level with significant differences are shown graphically in Supplementary Figures S4, S5. We also performed a corresponding analysis between fecal microbiota composition and the protective effect of D-galactose as shown in Supplementary Figure S6. Firmicutes and F/B ratio were positively correlated with the AD clinical symptoms index and the serum total IgE level. At the genus level, the relative abundance of *Bacteroides* had a positive correlation, and the relative abundance of *Prevotella* and *Ruminococcus* had a negative correlation with the clinical symptoms, consistent with the above description. Taken together, these results indicate that gut microbiota changed by D-galactose intake induce improvement of AD symptoms.

## Discussion

Atopic dermatitis is a chronic disease with a complicated immune response. Although typical steroid agents or anti-histamine agents are used as the first-line treatment for AD (26), these agents are associated with several side effects during long-term therapy (27). Therefore, alternative approaches for AD management are of increasing interest internationally. Recently, the role of intestinal microbiota has attracted the attention as studies reported its relationship with various diseases such as IBD, obesity and AD. The interaction between intestinal microbiota and host has led to interest in using alternative therapies for various diseases including AD by modulating microbiota.

In our study, oral administration of D-galactose improved AD in mice. Clinical symptoms of DNCB-induced AD-type lesions were alleviated by D-galactose administration. Histologic findings suggest that epithelial hyperplasia was decreased by D-galactose administration. In addition, accumulation and infiltration of immune cells, including mast cells, were suppressed in cutaneous tissues. We also found that D-galactose therapy modulates the diversity and composition of the intestinal microbiota.

D-galactose is widely distributed in biological systems and can be utilized by living organisms *via* the Leloir reaction (28, 29). It is known that D-galactose affects the profile of microbiota. Stahel et al. (20) reported that a daily intake of 15% galactose decreased the Firmicutes/Bacteroidetes (F/B) ratio compared with a diet of glucose and fructose in SD rats. Galacto-oligosaccharides, the galactose polymers used as a prebiotic, increased the growth of specific gut microbiota, resulted in immune modulation, and production of SCFAs (30). Accordingly, we hypothesize that D-galactose may alleviate the effect on AD by altering intestinal microbiota.

We found that administration of D-galactose induced alteration of intestinal microbiota. The diversity analysis showed that the bacterial diversity of the intestinal microbiota was upregulated in D-galactose-treated group compared with PBS group (Figures 5A,B). The relationship between AD and microbiota diversity is disputed. Lee et al. (31) reported that prenatal exposure to antibiotics and birth *via* C-section with a lower microbiota diversity increased the risk of AD. In contrast, Nylund et al. (32) reported that children with eczema showed higher diversity of intestinal microbiota compared with control subjects. Nonetheless, these studies have suggested that the altered diversity affected the development of allergic diseases. Based on composition analysis, the administration of D-galactose did not dramatically alter the microbiota in the absence of AD. Interestingly, in the presence of AD, D-galactose restored the changes in microbiota by increasing Bacteroidetes and decreasing Firmicutes at the phylum level. Intestinal microbiota are classified into two major phyla, Firmicutes and Bacteroidetes (33). Generally, the F/B ratio plays a key role in intestinal microbiota homeostasis. The altered ratio is dysbiosis, which is closely associated with various diseases (33). In previous clinical studies, the healthy group showed a different microbiota composition compared with the allergic disease group. In the healthy group, the relative abundance of Firmicutes was decreased from birth to adulthood. However, in the allergic disease group, the Firmicutes showed an increase following birth (34). These re-constructive changes in Firmicutes phylum composition may be related to allergic disease. The relative abundance of Bacteroidetes in the AD pregnant mothers was low and cesarean section delivery which is related to an increased risk of AD was associated with delayed colonization and the α-diversity of Bacteroidetes (35). The F/B ratio is recognized as an important marker of AD prevalence and F/B ratio reduction may be a mitigation mechanism of D-galactose against to AD.

Further, we analyzed metagenome sequencing in the class, order and family levels and added these results to the Supplementary Data. D-galactose intake restored Alphaproteobacteria and Deltaproteobacteria which are up-regulated and down-regulated respectively in DNCB-treated mice. Similarly, at the order level, Kiloniellales, an Alphaproteobacteria, and Desulfovibrionales, a Deltaproteobacteria, were restored respectively by D-galactose intake. Yang et al. (36) reported that the amount of Alphaproteobacteria EVs (Extracellular vesicles) was decreased in AD patients' serum. Myles et al. (37) also revealed that an increase in the Alphaproteobacteria of the skin microbiota composition was associated with *R. mucosa* treatment which improved of AD symptoms. However, to clarify the function of Alphaproteobacteria and Deltaproteobacteria in the gut microbiota, further studies are needed. At the family level, Arildsen et al. (38) reported that mice that failed to colonize Rikenellaceae were associated with a high-responding phenotype of oxazolone-induced dermatitis. In our results, D-galactose intake increased the abundance of Rikenellaceae in contrast to the PBS intake in DNCB-induced AD model, which is consistent with the previous report. At the sub-classification below the phylum level, metagenome sequencing analysis results suggested the protective effect of D-galactose intake against to AD.

In genus level, administration of D-galactose decreased the relative abundance of *Bacteroides* and increased the relative abundance of *Prevotella* and *Ruminococcus*. Previous studies reported that the AD group showed a higher relative abundance of *Bacteroides* (39, 40). *Bacteroides* spp. are the predominant human intestinal microbiota. However, their increased abundance has been associated with type 2 immune manifestations such as food allergy and atopic diseases. Indeed, the increased abundance of *Bacteroides* in AD triggers a continuous inflammatory response by production of lipopolysaccharide, the dominant component of the outer membrane of gram-negative bacteria (41). Laigaard et al. reported a significant correlation of clinical scores, serum IgE level and ear tissue cytokine levels with the abundance of *Prevotella* spp. in oxazolone-induced AD mice. The correlation between the ameliorated phenotype and increased abundance of *Prevotella* spp. in mice indicates its protective role in allergic immune response (42). The proportion of *Ruminococcus* spp. was also associated with AD. Kwon et al. reported that *Ruminococcus* spp. were significantly decreased in DNCB-induced AD mice. However, these bacteria were restored by treatment with *L. sakei* WIKIM 30. It was shown that abundance of *Ruminococcus* may be correlated with the regulatory T cell-related response (43). Likewise, intestinal microbiota modulation by D-galactose administration may facilitate the protective immune response to AD in mice. However, the mechanism underlying the role of D-galactose in altering intestinal microbiota structure remains to be determined. We conducted an analysis based on OTUs, which have been used in many studies previously. However, with the recent development of an algorithm that can accurately detect sequence mutations while reducing errors, the analysis of ASV instead of OTUs analysis has emerged. It is considered that microbiota data based on the ASV classification system should be accumulated in further studies on the relationship between gut microbiota and AD.

The microbiota play a pivotal role in the formation and development of the host's innate and adaptive immune system (44). The activities of microbiota depend strongly on effective communication with other bacteria and hosts. Quorum sensing (QS) is the representative communication system between bacteria and the host (45). QS can induce alteration in bacterial gene expression and bacterial biofilm formation (46). This communication can be altered by numerous biomolecules ranging from nutrients to signaling molecules (45). Especially, autoinducer-2 (AI-2) is one of the common QS signaling molecules. Most of bacteria can respond to AI-2 and regulate their attachment, motility and biofilm formation (47). These bacteria have a close relationship with host cells, which induce changes in intestinal microbiota (48). Thompson et al. (49) reported that AI-2 signaling modulates microbiota composition *via* alteration in the concentration of metabolites within the gut and downstream effect on host physiology and immunology. Interestingly, Ryu et al. (50) reported that D-galactose suppressed the biofilm formation of periodontal pathogens by inhibiting AI-2 activity. D-galactose showed high sequence similarity between D-galactose-binding protein and AI-2 receptor (50). These studies suggest that the effect of D-galactose intake *via* inhibition of AI-2 signaling may influence biofilm formation of intestinal bacteria and alter the QS signaling. It is expected that these mechanisms alleviate AD symptoms *via* microbiota modulation following D-galactose administration. Further studies are required to evaluate whether the intestinal microbiota modified by D-galactose intake is related to AI-2 signaling and biofilm formation. In addition, D-galactose can be converted to glucose-6-phosphate (G-6-P) in the liver through several enzymatic steps (51). However, to our knowledge, there is no evidence to show the direct effect of G-6-P or intermediate metabolites in AD development. A further study is needed to clarify a correlation between the metabolites of D-galactose and AD development.

## Conclusion

In summary, our results demonstrated that D-galactose has a protective effect on DNCB-induced atopic dermatitis *via* suppression of serum IgE levels leading to anti-inflammatory effects by modulating the intestinal microbiota. Modulation of microbiota *via* D-galactose restores bacterial diversity and bacterial composition, suggesting an alternative therapeutic strategy against AD.

## Data Availability Statement

The datasets presented in this study can be found in online repositories. The names of the repository/repositories and accession number(s) can be found in online site: https://www.ebi.ac.uk/arrayexpress/, E-MTAB-11632.

## Ethics Statement

The animal study was reviewed and approved by the Institutional Animal Care and Use Committee of Chonnam National University (Gwangju, Korea; approval no. CNU IACUC-YB-2020-68).

## Author Contributions

J-HP, D-YK, and D-HJ contributed substantially to the conception of the work. D-YK and D-HJ made substantial contribution to experimental work, data analysis and manuscript writing. E-JS, A-RJ, J-YP, J-HA, T-SL, Y-JK, Y-JL, and I-SS performed animal experiments. T-SL, Y-JK, H-EK, and E-JR performed histological analysis. JS wrote manuscript. All authors approved the final version of this manuscript to be published and agree to be accountable for all aspects of the work in ensuring that questions related to the accuracy or integrity of any part of the work are appropriately investigated and resolved.

## Funding

This research was funded by Quorum Bio Co., Ltd., School of Dentistry, Seoul National University, Seoul, South Korea (Contract No. 2020-3417). The authors declare that this study received funding from Quorum Bio Co., Ltd. The funder was not involved in the study design, collection, analysis, interpretation of data, the writing of this article or the decision to submit it for publication.

## Conflict of Interest

H-EK, E-JR, and JS were employed by Quorum Bio Co., Ltd. The remaining authors declare that the research was conducted in the absence of any commercial or financial relationships that could be construed as a potential conflict of interest.

## Publisher's Note

All claims expressed in this article are solely those of the authors and do not necessarily represent those of their affiliated organizations, or those of the publisher, the editors and the reviewers. Any product that may be evaluated in this article, or claim that may be made by its manufacturer, is not guaranteed or endorsed by the publisher.
