# Supplementary material for: D-galactose Intake Alleviates Atopic Dermatitis in Mice by Modulating Intestinal Microbiota
Source: Front Nutr. 2022 Jun 21;9:895837. doi: 10.3389/fnut.2022.895837 (PMC9254681; doi:10.3389/fnut.2022.895837)
Supplement: Supplementary file 1 [file Data_Sheet_1.DOCX]

Supplementary Material

# Supplementary Data


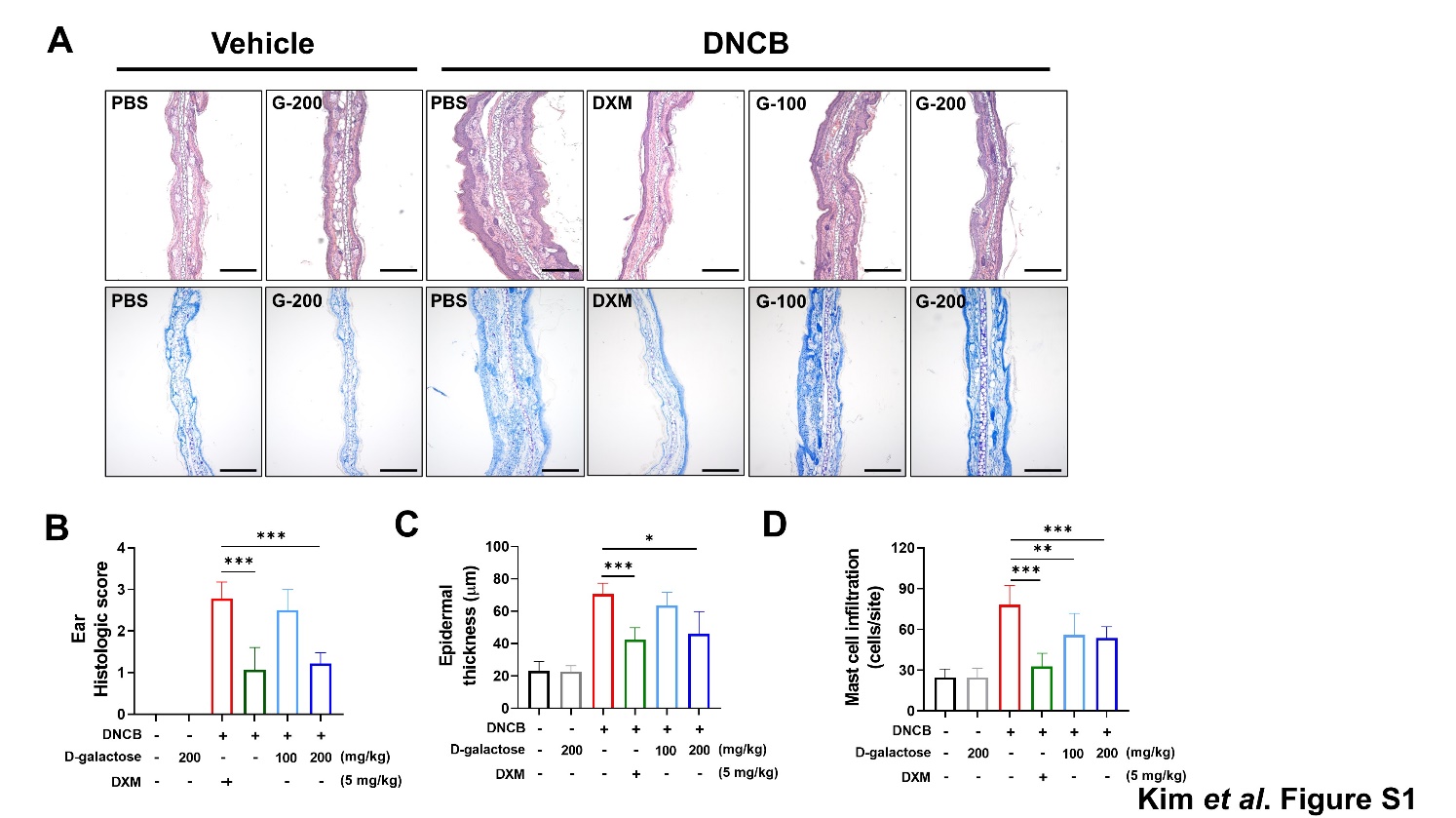


**Supplementary Figure 1. Effects of D-galactose on ear skin lesions.**

Representative histological findings of ear skin sections stained with **(A)** hematoxylin and eosin or toluidine blue staining (Scale bars, 100 μm). Histological evaluation of **(B)** ear skin sections and **(C)** epidermal thickness. **(D)** Mast cell infiltration was quantified and compared among the groups. Values are means ± SD. The significance of differences between the group was assessed using Mann-Whitney U-test, with the level of significance set at **p* < 0.05, ***p* < 0.01, ****p* < 0.001.
