# Supplementary material for: D-galactose Intake Alleviates Atopic Dermatitis in Mice by Modulating Intestinal Microbiota
Source: Front Nutr. 2022 Jun 21;9:895837. doi: 10.3389/fnut.2022.895837 (PMC9254681; doi:10.3389/fnut.2022.895837)
Supplement: Supplementary file 2 [file Data_Sheet_2.DOCX]

Supplementary Material

# Supplementary Data


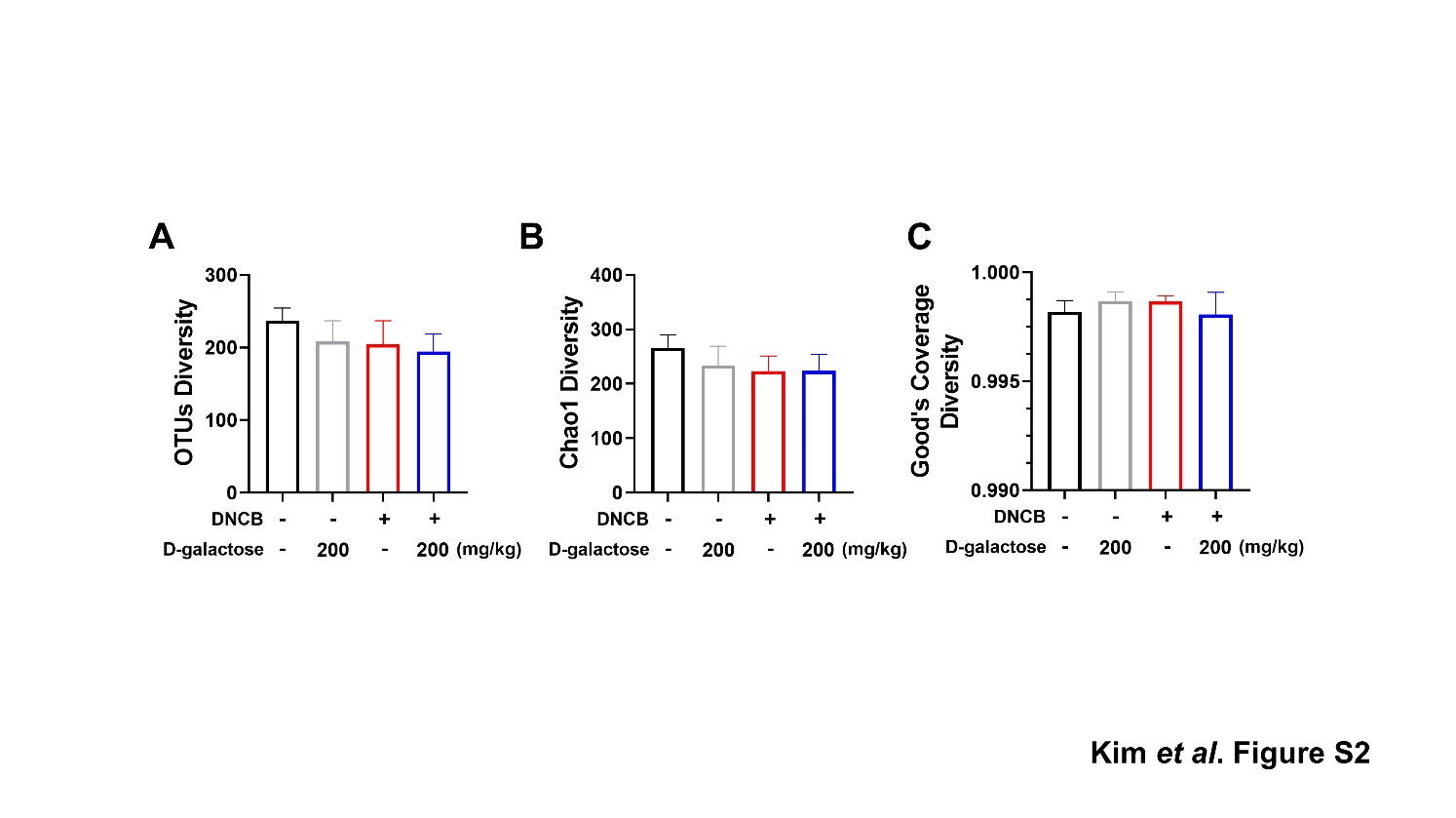


**Supplementary Figure 2.** **Effects of D-galactose on intestinal microbiota alpha diversity of DNCB-induced atopic dermatitis in mice**

**(A-C)** Alpha diversity determined by OTUs, Chao1, Good’s coverage diversity. Values are means ± SD. The significance of differences between the group was assessed using Mann-Whitney U-test.
