# Supplementary material for: D-galactose Intake Alleviates Atopic Dermatitis in Mice by Modulating Intestinal Microbiota
Source: Front Nutr. 2022 Jun 21;9:895837. doi: 10.3389/fnut.2022.895837 (PMC9254681; doi:10.3389/fnut.2022.895837)
Supplement: Supplementary file 3 [file Data_Sheet_3.DOCX]

Supplementary Material

# Supplementary Data


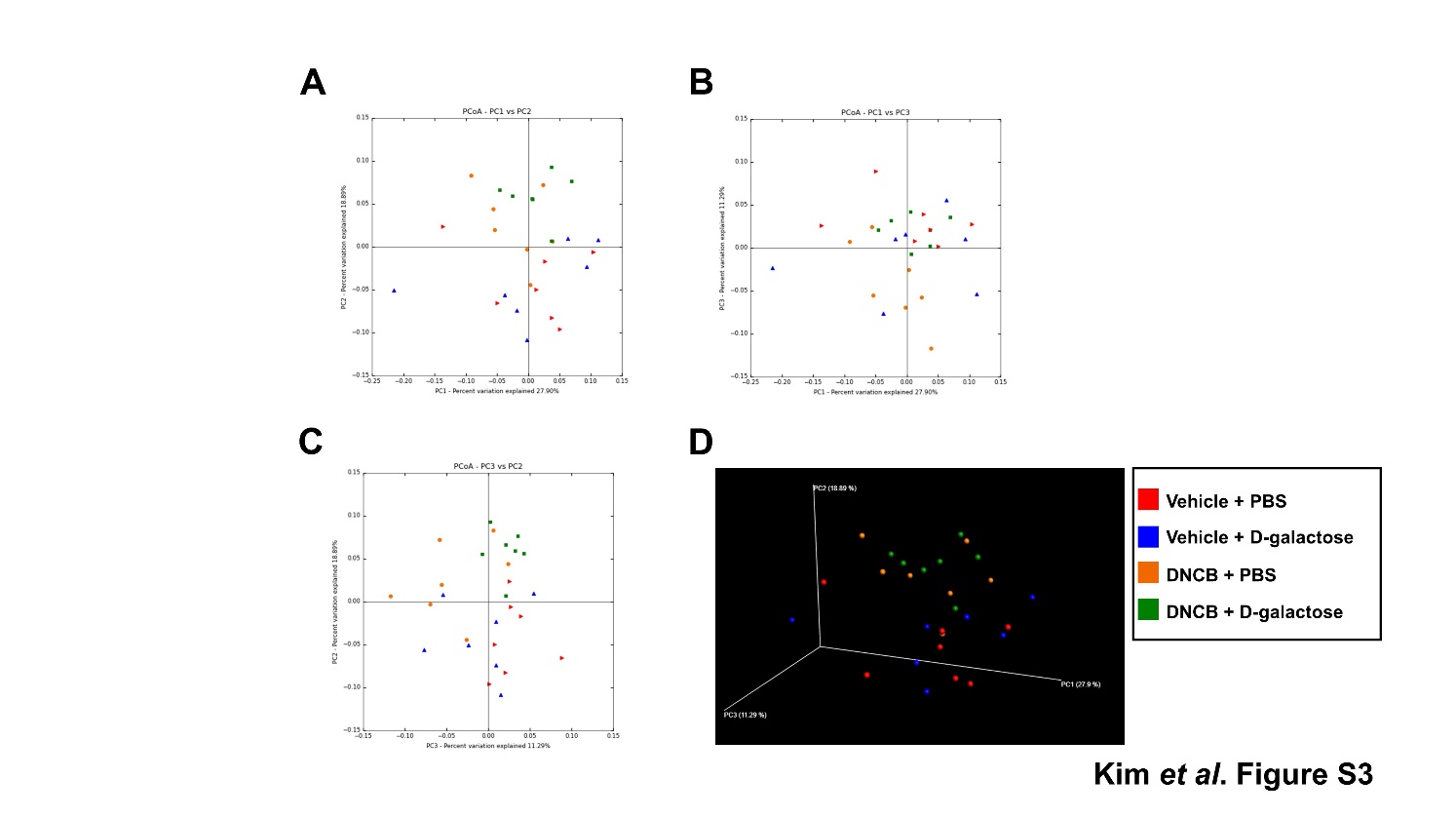


**Supplementary Figure 3. Effects of D-galactose on intestinal microbiota beta diversity of DNCB-induced atopic dermatitis in mice**

**(A-D)** Beta diversity 2D, 3D pattern determined by weight principal coordinate analysis., PBS + Vehicle (Red), D-Galactose + Vehicle (Blue), PBS + DNCB (Yellow), D-Galactose + DNCB (Green)
