# Supplementary material for: D-galactose Intake Alleviates Atopic Dermatitis in Mice by Modulating Intestinal Microbiota
Source: Front Nutr. 2022 Jun 21;9:895837. doi: 10.3389/fnut.2022.895837 (PMC9254681; doi:10.3389/fnut.2022.895837)
Supplement: Supplementary file 5 [file Data_Sheet_5.DOCX]

Supplementary Material

# Supplementary Data


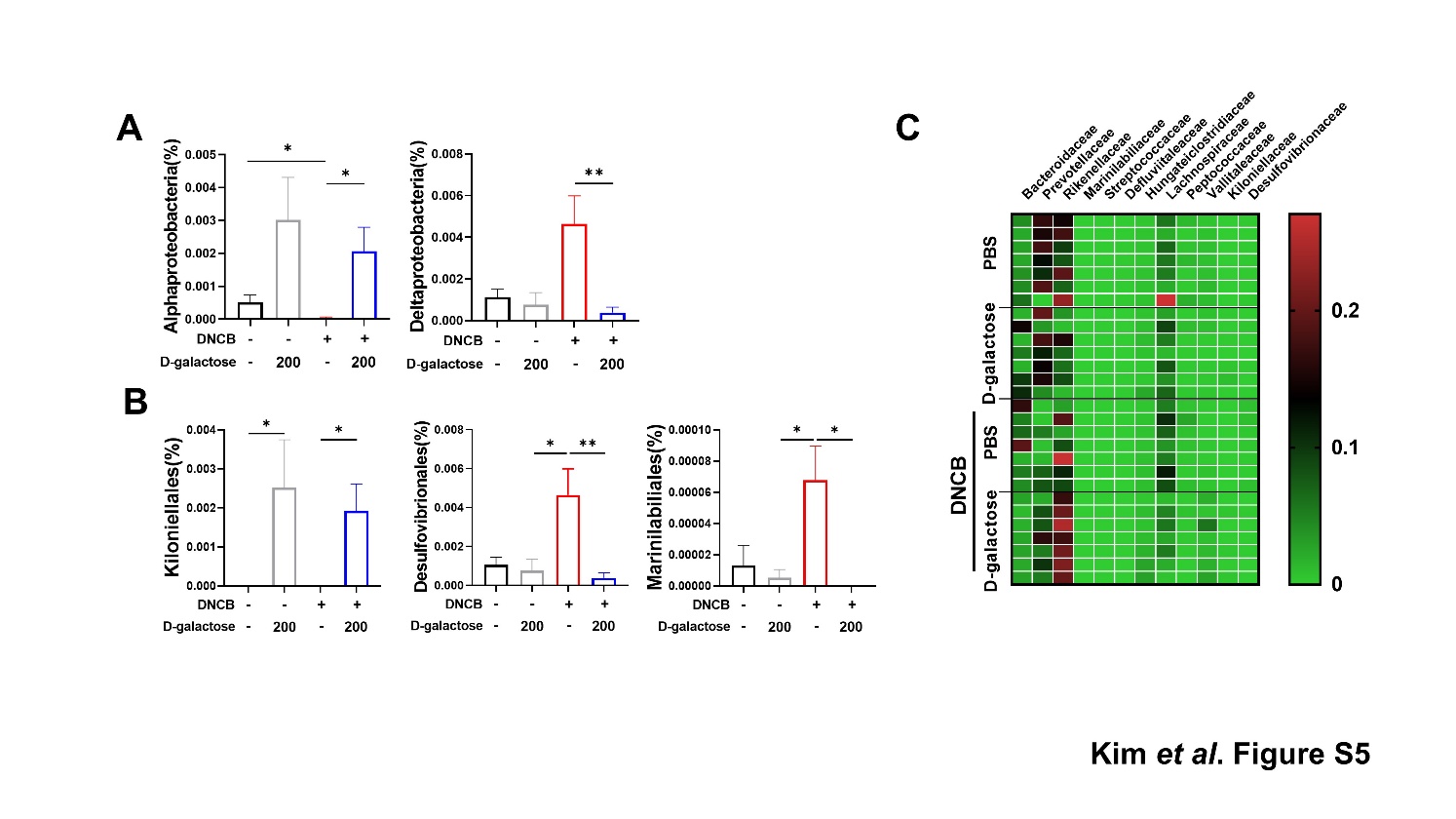


**Supplementary Figure 5. Effects of D-galactose on intestinal microbiota compositions of DNCB-induced atopic dermatitis in mice in class, order and family level**

**(A)** Relative abundance of intestinal microbiota in Class level which is significantly difference between DNCB-induced AD group. **(B)** Relative abundance of intestinal microbiota in Order level which is significantly difference between DNCB-induced AD group. **(C)** Relative abundance of intestinal microbiota in Family level which is significantly difference between DNCB-induced AD group. Values are means ± SD. The significance of differences between the group was assessed using Mann-Whitney U-test, with the level of significance set at **p* < 0.05, ***p* < 0.01.
