# Supplementary material for: D-galactose Intake Alleviates Atopic Dermatitis in Mice by Modulating Intestinal Microbiota
Source: Front Nutr. 2022 Jun 21;9:895837. doi: 10.3389/fnut.2022.895837 (PMC9254681; doi:10.3389/fnut.2022.895837)
Supplement: Supplementary file 6 [file Data_Sheet_6.DOCX]

Supplementary Material

# Supplementary Data


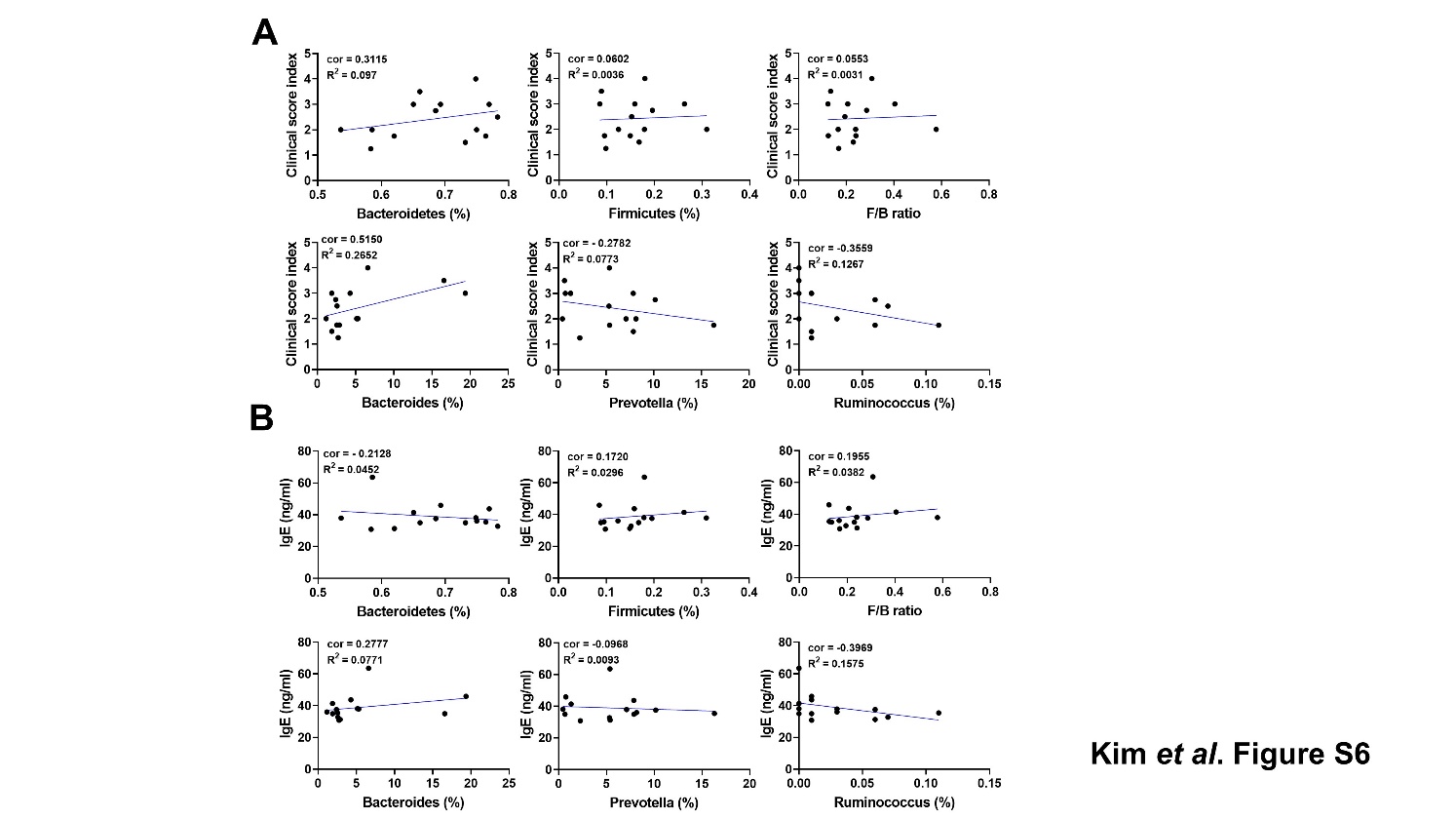


**Supplementary Figure 6. Correlation between the gut microbiota and key AD parameters.**

**(A)** Correlation between gut microbiota composition and Clinical score index. **(B)** Correlation between gut microbiota composition and serum IgE. Cor: Correlation matrix (Pearson), R^2^: Coefficients of determination
